# Assessing palliative care needs in adult patients with hematological malignancies and their caregivers: implications for referral practice

**DOI:** 10.1186/s12904-025-01811-5

**Published:** 2025-07-14

**Authors:** Radhika R. Pai, Arun Ghoshal, Karthik Udupa, Ananth Pai, Sharada Mailankody, Anuja Damani, Krithika S. Rao, Shwetha Prabhu, Malathi G. Nayak, Naveen Salins

**Affiliations:** 1 Department of Fundamentals of Nursing, Manipal College of Nursing, Manipal Academy of Higher Education, Manipal, Karnataka 576104 India; 2 Department of Palliative Medicine and Supportive Care, Kasturba Medical College Manipal, Manipal Academy of Higher Education, Manipal, Karnataka 576104 India; 3 Manipal College of Nursing, Manipal Academy of Higher Education, Manipal, Karnataka 576104 India; 4 Community Health Nursing Department, Manipal College of Nursing, Manipal Academy of Higher Education, Manipal, Karnataka 576104 India; 5 Department Medical Oncology Kasturba Medical College, Manipal, Manipal Academy of Higher Education, Manipal, Karnataka 576104 India

**Keywords:** Hematologic neoplasms, Palliative care, Needs assessment, Referral and consultation, Adult, Caregivers, Quality of life, Patient care needs, Psychological distress, Health services needs and demand, Supportive care, End-of-life care, Oncology nursing, Patient care planning, Health care surveys, Caregiver burden, Health services accessibility, Symptom management

## Abstract

**Background:**

Patients with hematological malignancies frequently experience complex physical, psychological, and spiritual concerns due to disease progression and intensive therapies. Despite these needs, palliative care services are often underutilized. This study aimed to assess the palliative care needs of adult patients with hematological malignancies and their caregivers in a tertiary care setting in India.

**Methods:**

A descriptive survey was conducted from October 2022 to February 2023, involving 200 patients and their caregivers in oncology wards. The Needs Assessment Tool for Progressive Disease-Cancer (NAT: PD-C) was used to evaluate physical, psychological, and social needs. Data were analyzed using descriptive statistics in Jamovi 2.0.0.

**Results:**

Of the 200 patients, 55.5% reported no unresolved physical symptoms, while 9.5% had significant concerns, including fatigue, pain, and breathlessness. Psychological distress was noted in 16% of patients, with 15.62% requiring referral to specialized services. Caregivers reported significant concerns regarding physical strain (2%) and psychological distress (14%). Only 9.55% of patients were referred to specialist palliative care services, highlighting a gap in timely palliative care access.

**Conclusions:**

Despite a high burden of symptoms, specialist palliative care services are underutilized for patients with hematological malignancies. Early integration of palliative care can improve symptom management, reduce psychological distress, and enhance patients’ and caregivers’ quality of life. Training healthcare providers in palliative care principles and establishing standardized referral pathways are essential to address these unmet needs.

**Supplementary Information:**

The online version contains supplementary material available at 10.1186/s12904-025-01811-5.

## Introduction

Hematological malignancies, comprising a diverse group of cancers such as leukemia, lymphoma, and multiple myeloma, pose significant challenges not only due to their complexity but also because of their impact on patients’ quality of life (QoL). These malignancies often have aggressive disease courses, necessitating intensive treatments like chemotherapy, radiation, or stem cell transplantation. While such treatments are essential for survival, they frequently lead to profound physical, psychological, and spiritual distress in patients, particularly as the disease progresses [1]. Despite this, palliative care remains underutilized in managing the symptoms and supportive care needs of patients with hematological cancers. This gap in care is particularly problematic, as palliative care has been shown to alleviate symptom burden and enhance QoL when integrated early in the disease trajectory [2, 3]. Curative treatment options are more readily available for hematological malignancies through various cancer treatment modalities, as compared to solid tumors [4]. 

In high-income countries, the integration of palliative care with curative treatments for solid tumors is now a widely accepted model. However, the uptake of palliative care services remains limited for hematological malignancies [5, 6]. Various barriers contribute to this underutilization, including a lack of awareness among healthcare professionals, patients, and caregivers about the potential benefits of palliative care in these cases [7]. Moreover, many hematologists prioritize disease-directed therapies over supportive care, often delaying palliative care referrals until very late in the disease process. This situation is exacerbated in low- and middle-income countries (LMICs) like India, where limited resources, inadequate training of healthcare professionals, and insufficient public awareness of palliative care further hinder its early integration [8]. 

Palliative care is defined by the World Health Organization (WHO) as an approach that improves the QoL of patients and their families facing life-threatening illnesses by preventing and alleviating suffering. This is achieved through early identification, comprehensive assessment, and the treatment of pain and other physical, psychosocial, and spiritual problems [9]. The scope of palliative care extends beyond end-of-life care, offering critical support from the point of diagnosis through treatment and, if necessary, into survivorship or the terminal phases of illness. Despite its documented benefits in terms of symptom management, communication, decision-making, and emotional support, patients with hematological malignancies are less likely to be referred to palliative care than those with solid tumors [10]. 

One reason for the delayed referral of hematological malignancy patients to palliative care is the perception that their disease trajectory is more unpredictable compared to solid tumors. Hematological cancers can follow a protracted course, often punctuated by periods of remission and relapse, which may lead clinicians to defer palliative care referrals, assuming that the need for supportive care will arise only in the terminal phase. Moreover, hematologists and oncologists may fear that referring patients to palliative care will convey a message of “giving up” on curative treatment, potentially undermining the patient’s hope [11]. Such misconceptions about palliative care being synonymous with end-of-life care limit its timely integration.

Studies have shown that patients with hematological malignancies experience a higher symptom burden than those with solid tumors, particularly with cytopenia-related symptoms like infection, fatigue, dyspnea, and bleeding [1]. In addition, they may suffer from severe psychological distress, including anxiety and depression, due to the intensity of treatment regimens and the uncertainty surrounding their prognosis [12]. However, despite these complex needs, palliative care is seldom introduced early enough to address these challenges effectively. Early palliative care intervention has been associated with better symptom management, improved patient satisfaction, and reduced psychological distress [10]. 

Another important consideration is the impact of hematological malignancies on caregivers. Family caregivers, who often play a central role in the daily management of patients with cancer, report high levels of physical, emotional, and financial strain. The unpredictability of hematological cancers, along with the intensive nature of treatments, places an immense burden on caregivers, who are frequently responsible for managing complex care needs at home [13]. Research has shown that caregiver burden is directly correlated with the patient’s symptom burden and overall health status, highlighting the need for comprehensive palliative care services that address both patient and caregiver needs [14]. By offering counseling, respite care, and practical support, palliative care can significantly alleviate the stress experienced by caregivers.

The Needs Assessment Tool for Progressive Disease—Cancer (NAT: PD-C), employed in this study, provides a systematic framework for evaluating the palliative care needs of patients and their caregivers [15]. This tool has been validated in various settings, including general practice and oncology, and has proven effective in identifying unmet needs across physical, psychological, and social domains. It guides healthcare professionals in determining the appropriate level of care, including whether referral to specialized palliative care services is necessary. Although the NAT: PD-C has been primarily used in high-income settings, its application in LMICs, such as India, can potentially optimize the allocation of limited healthcare resources and ensure that patients and caregivers receive the support they require [16]. 

This study aims to address the existing gap in palliative care for patients with hematological malignancies by evaluating their unmet needs and those of their caregivers. By assessing these needs using a validated tool, the study provides valuable insights into the barriers to palliative care referral and utilization in a tertiary care hospital in India.

## Methods

This study utilized a descriptive survey design to evaluate the palliative care needs of adult patients diagnosed with hematological malignancies and their caregivers. The research was conducted in the oncology wards of a tertiary care hospital in India. All consecutive patients accompanied by their caregivers were approached between October 2022 and February 2023. According to hospital rules, all patients who visit for daycare, chemotherapy, or hospital admission must have a caregiver present. Ethical approval for the study was granted by the Institutional Ethics Committee of Kasturba Medical College and Kasturba Hospital in Manipal (IEC1-82/2022). Written informed consent was obtained from all participants before their inclusion in the study, ensuring compliance with ethical standards and respecting patient autonomy.

The study population consisted of 200 patients diagnosed with various hematological malignancies, including leukemia, lymphoma, and multiple myeloma, and their caregivers. Patients were included based on an enumerative sampling technique, ensuring that all eligible patients admitted to the oncology wards during the study period were invited to participate. The inclusion criteria were adult patients aged 18 years and older with a confirmed diagnosis of hematological malignancy. Caregivers, individuals providing primary informal care to the patient, were also included in the assessment to capture their palliative care needs. All the patients who come to the hospital for either daycare chemotherapy or hospital admission were accompanied by a caregiver as per the hospital rules.

Data were collected using two main tools: a demographic proforma and the Needs Assessment Tool for Progressive disease—cancer (NAT: PD-C) (please see Supplement I). The demographic proforma captured patient and caregiver information, including age, gender, marital status, educational level, and diagnosis. The NAT: PD-C is a comprehensive assessment tool that identifies the palliative care needs of patients and their caregivers by evaluating symptoms, psychological well-being, daily living activities, and other care domains (Waller et al., 2010). The tool has been validated for use in oncology and general practice settings. It is divided into four sections: (1) priority referral for further assessment, (2) patient well-being, (3) caregiver’s ability to care for the patient, and (4) caregiver well-being.

Each section of the NAT: PD-C includes specific questions designed to assess the level of concern (none, some/potential, or significant) and the action taken (directly managed, managed by other care team members, or referral required). In this study, the action taken is by the consultant medical oncologist and this portion of the tool was completed by checking the records for the treatment or referral details for a particular problem expressed by the patient. This structured approach enables healthcare professionals to identify the most pressing needs and determine whether specialist palliative care referral is warranted. In this study, data were collected by the trained healthcare professional using an interview technique, ensuring accurate and consistent use of the NAT: PD-C tool. Level of concern as per the patient report is documented as per the significant effect in the patient’s life, and those who expressed some/potential as well as significant concern were asked to report the action taken for addressing the same. Validation of this tool was done with 5 subject experts, and the tool is found to be reliable (Content Validity Index = 0.9). The tool was translated into the local language, Kannada. The tool was retranslated into English to verify accuracy and identify potential errors.

The primary outcome of interest was identifying unmet palliative care needs in patients and caregivers. Secondary outcomes included the proportion of patients and caregivers referred to specialist palliative care services and the identification of factors associated with the underutilization of these services. Descriptive statistical methods were used to analyze the data, including frequency distributions, percentages, and cross-tabulations. The data analysis was conducted using Jamovi 2.0.0 software, which facilitated the identification of trends and patterns in responses.

## Results

Out of around 239 approached, 200 patients and caregivers with hematological malignancies consented to participate in the study. The demographic characteristics of the patients revealed that the majority, 59(29.5%), were aged between 58 and 67 years, with 32(16%) being older than 68 years. Regarding gender distribution, 69(34.5%) participants were female, while the remaining 131(65.5%) were male. Most patients 164(82%) were married, and 146(73%) had completed primary education. The most common diagnosis among the patients was leukemia, affecting 76(38%), followed by lymphoma, 72(44%), and myeloblastic malignancy, which was diagnosed in 26(52%) of patients (please see Table 1).

**Table 1:** Demographic and clinical proforma, *n* = 200

Items		Numbers	Percentage (%)
**Age in years**	18–27 28–37 38–47 48–57 58–67 68+	12 20 26 51 59 32	6 10 13 25.5 29.5 16
**Gender**	Male Female	131 69	65.5 34.5
**Marital status**	Married Separated/divorced. Single widowed	164 1 21 14	82.0 0.5 10.5 7.0
**Education**	Primary school Secondary school Certificate/diploma University degree	146 24 13 17	73 12 6.5 8.5
**Type of hematological malignancy**	Leukemias		
	Acute	61	30.5
	Chronic	15	7.5
	Lymphomas	72	36
	Myelomas	52	26

The NAT: PD-C tool assessed patients’ and caregivers’ physical, psychological, and social concerns. In terms of physical symptoms, 111(55.5%) of patients reported no unresolved symptoms, while 70(35%) experienced some level of physical discomfort, and 19(9.5%) reported significant unresolved physical symptoms, particularly fatigue, pain, and breathlessness. Notably, 60(67.42%) of patients with physical symptoms were managed directly by their oncology team, while 19(21.35%) required referral to other departments, such as radiation oncology or specialist palliative care (please see Table 2).

Regarding the patient’s psychological well-being, 168(84%) reported no psychological distress, 26(13%) experienced mild symptoms of anxiety or depression, and 6(3%) reported significant psychological distress. Among those with psychological symptoms, 27(84.37%) were managed directly by their oncology consultants, while 5(15.62%) required referrals to specialized mental health services. In contrast, only 100(5%) of patients reported spiritual or existential concerns, all managing these concerns independently, without requiring external support. Concerning the information needs expressed by the patients, it was reported that most of them required information on medical treatment and psychological services.

**Table 2:** Description of NAT: PD-C, *n* = 200 sect. 1: priority referral for further assessment

Section 1: Priority referral for further assessment						
Content					Yes (%)	No (%)
Does the patient have a caregiver readily available if required?					193 (96.5)	7(3.5)
Has the patient or caregiver requested a referral to a specialist palliative care service (SPCS)?					15 (7.5)	185 (92.5)
Do you require assistance in managing the care of this patient and family?					51 (25.5)	149 (74.5)

Section 2: Patient Wellbeing						
Content	Level of concern			Action taken		
	None (%)	Some/ potential (%)	Significant (%)	Directly managed (%)	Managed by other care team members (%)	Referral required (%)
Physical symptoms	111	70	19	60 (67.42)	10(11.23)	19 (21.35)
(Unresolved)	(55.5)	(35)	(9.5)			
Appetite	-	-	2			
Bowel	-	-	1			
Breathlessness	-	3	10			
Cough	-	4	2			
Edema	-	-	3			
Fatigue	-	6	16			
Nausea	-	-	2			
Pain	-	13	21			
Sleeping	-	1	5			
Problems with Activities of daily living	141	36	23	53	-	6
	(70.5)	(18)	(11.5)	(89.8)		(10.2)
Psychological symptoms interfering with wellbeing	168 (84)	26	6	27	-	5
		(13)	(3)	(84.37)		(15.62)
Concerns about spiritual/Existential issues	195	5	-	5	-	-
	(97.5)	(2.5)		(100)		
Concerns about Financial/Legal causing distress	64	11	125	128	-	8
	(32)	(5.5)	(62.5)	(94.11)		(5.88)
Health Beliefs, Social and Cultural factors making care more complex	199 (99.5)	1	-	1	-	-
		(0.5)		(100)		
Does the patient have concerns about managing their medication and treatment regimens?	173	24	3	27	-	-
	(86.5)	(12)	(1.5)	(100)		
Does the patient have concerns about their sexual functioning or relationship?	200	-	-	-	-	
	(100)					
**Information Needs**						
**Does the patient require information? (tick any options that are relevant)**					**Information need**	
					**(Frequency)**	
Financial and legal assistance					33	
Psychological services					96	
Diagnosis					2	
Medical/health/support services					95	
Prognosis					1	
Treatment options					1	
**Section 3: The ability of the caregiver or family to care for the patient, n = 200**						
**Content**	**Level of concern**			**Action taken**		
	**None** **n (%)**	**Some/ potential** **n (%)**	**Significant** **n (%)**	**Directly managed.** **n (%)**	**Managed by other care team members n (%)**	**Referral required.** **n (%)**
Physical symptoms causing caregiver and family distress?	150 (75)	46 (23)	4(2)	48 (96)	-	2(4)
Providing physical care						
Is the caregiver having difficulty coping with activities of daily living	167 (83.5)	23 (11.5)	8 (4)	23 (74.2)	-	8 (25.8)
**Section 4: Caregiver Wellbeing**						
**Content**	**Level of concern**			**Action taken**		
	**None (%)**	**Some/ potential (%)**	**Significant (%)**	**Directly managed (%)**	**Managed by other care team members (%)**	**Referral required (%)**
Difficulty in coping with medical regimes	172 (86)	26 (13)	2 (1)	28(100)	-	-
Is the caregiver having difficulty coping with the patient’s psychological symptoms?	152 (76)	44 (22)	4 (2)	44 (91.66)	-	4 (26.6)
Caregivers have financial or legal concerns causing distress	194 (97)	5 (2.5)	1 (0.5)	2 (33.3)	-	4 (66.6)
Information needs on the course and prognosis of the disease and treatment	124 (62)	48 (24)	28 (14)	55 (72.36)	-	21(27.63)
**Content**	**Level of concern**			**Action taken**		
	**None** **n (%)**	**Some/** **potential n (%)**	**Significant n (%)**	**Directly managed n (%)**	**Managed by other care team members n (%)**	**Referral required n (%)**
Caregiver experiencing physical, practical, spiritual, existential, or psychological problems that interfere with their well-being/functioning.	124 (62.2)	54 (27)	22(11)	76 (97.43)	-	2 (2.56)
Caregiver experiencing grief over the impending or recent death that is interfering with well-being/functioning	166 (98)	32 (16)	2 (1)	34(100)	-	-

Table 3 outlines referral details for patients, highlighting both the types of specialists involved and the urgency of assessments post-completion of the Needs Assessment Tool: Progressive Disease – Cancer (NAT: PD-C). Most referrals were made to radiation oncologists, 56(35.67%), followed by medical oncologists, 38(24.20%). A significant portion of patients, 88 (56.05%), were deemed non-urgent, with only 14.01% requiring urgent attention within 24 h. All referrals were discussed with the patients, and every patient, 157(100%), gave consent to proceed with further consultation.

**Table 3:** Referral details, *n* = 157

Referral details		*n*	%
Referral to: (Specialty)	General practitioner Psychologist Specialist palliative care service Medical oncologist Radiation oncologist	23 25 15 38 56	14.65 15.92 9.55 24.20 35.67
Priority of assessment needed	Urgent (within 24 h) Semi-Urgent (2–7 days) Non-urgent (next available)	22 47 88	14.01 29.94 56.05
Discussed the referral with the client.	Yes No	157 0	100 0
The client consented to the referral.	Yes No	157 0	100 0

## Discussion

This study provides valuable insights into the palliative care needs of patients with hematological malignancies and their caregivers in a tertiary care setting in India. The results demonstrate that, despite the high burden of physical and psychological symptoms experienced by these patients, palliative care referrals remain significantly underutilized. Only 9.55% of patients in this study were referred to specialist palliative care services, and 35% (*n* = 70) expressed some/potential symptoms that were not addressed, highlighting a critical gap in the care continuum for hematological malignancy patients. These findings are consistent with previous studies that have identified barriers to palliative care referrals for this population, including misconceptions about the role of palliative care, the unpredictable disease trajectory of hematological malignancies, and the curative focus of oncologists and hematologists [2, 17]. Another study supported this well, which stated challenges associated with referrals, trust issues, power relationships, and presuppositions as obstacles to palliative care referral among hematologists and oncologists [6]. At least one unmet need was reported by 95% (60/63), and the mean number of unmet needs per patient was 7 (0–18) in a study conducted in India using CaSUN (Cancer Survivors Unmet Needs Measure) among 63 multiple myeloma patients [18]. 

One of the most notable findings from this study is the significant symptom burden reported by patients, particularly concerning pain, fatigue, and breathlessness. These symptoms are well-documented in the literature as being common among patients with hematological malignancies, and they often persist throughout the course of the disease, even in patients receiving curative treatments [19]. However, despite the prevalence of these symptoms, the referral to specialist palliative care services was low, with most patients’ symptoms managed by their oncology team. This suggests that there may be a reluctance among oncologists and hematologists to refer patients to palliative care, possibly due to concerns that doing so may signal a shift away from curative intent [11, 20]. 

Another significant finding is the psychological distress experienced by patients and caregivers. Although 84% of patients reported no psychological symptoms, a considerable minority (16%) experienced mild to significant distress, including anxiety and depression. Caregivers, too, reported significant distress, with 13% expressing concerns about managing the patient’s psychological symptoms and 14% reporting significant distress related to uncertainty about the patient’s prognosis and treatment options. These findings underscore the need for psychological support services to be integrated into the palliative care framework for patients with hematological malignancies, particularly given the complex nature of these diseases and their treatments [12]. 

The low referral rate to specialist palliative care services in this study is concerning, particularly given the demonstrated benefits of early palliative care integration for patients with hematological malignancies. Research has shown that early palliative care improves symptom management, enhances patient satisfaction, reduces psychological distress, and even improves survival outcomes in some cases [10]. Moreover, early palliative care allows patients and their families to engage in advance care planning, make informed decisions about their treatment options, and receive support for complex symptom management and emotional needs [2]. The findings from this study suggest a pressing need for oncologists and hematologists to receive more training in palliative care principles and to understand the value of integrating palliative care early in the disease trajectory. We, however, did not explore this area in detail and it is a limitation of this research work.

The caregiver burden was another important focus of this study. Caregivers play a central role in managing the day-to-day care of patients with hematological malignancies, often taking on significant physical and emotional responsibilities. In this study, 24% of caregivers expressed a need for more information about the patient’s prognosis and treatment options, while 14% reported significant distress related to the uncertainty of the course. These findings are consistent with previous research that has identified unmet information and emotional support needs as significant sources of distress for caregivers of cancer patients [21]. By addressing these needs through early palliative care referrals and providing caregivers with practical support, healthcare providers can help alleviate caregiver burden and improve patient and caregiver outcomes.

At any point during the course of the hematologic malignancy, hemato-oncologists and palliative care teams can effectively collaborate to meet the diverse requirements of cancer patients and their families. According to high-quality evidence, early palliative care team involvement has been shown to improve the quality and duration of life for cancer patients [22]. There is a need for novel palliative care and cancer care integration models [23]. 

This study also has implications for clinical practice in LMICs, where access to specialist palliative care services is often limited. In India, where this study was conducted, palliative care services are still in the early stages of development, and many healthcare providers lack training in palliative care principles [24]. Resource constraints and a lack of public awareness about the benefits of palliative care compound this situation. The findings from this study highlight the need for systemic changes in how palliative care is delivered in LMICs, particularly for patients with hematological malignancies, who may have complex and ongoing care needs. By implementing standardized referral pathways and training healthcare providers in palliative care, it may be possible to improve access to these services and ensure that patients and caregivers receive the support they need.

## Conclusion

This study highlights significant unmet palliative care needs among patients with hematological malignancies and their caregivers, with low referral rates to specialist palliative care services despite a high symptom burden. Early integration of palliative care is crucial for improving symptom management, reducing psychological distress, and enhancing patients’ and caregivers’ quality of life. There is an urgent need for healthcare providers, particularly in low- and middle-income countries, to receive training in palliative care principles. Standardized referral protocols can help ensure that these vulnerable populations receive timely, comprehensive, and supportive care throughout their illness trajectory.

## Electronic supplementary material

Below is the link to the electronic supplementary material.

Supplementary Material 1

## Data Availability

The datasets used and/or analyzed during the current study are available from the corresponding author upon reasonable request.

## Abbreviations

NAT: PD-C: Needs assessment tool: progressive disease – cancer
ASR: Age-adjusted rate
SPC: Specialist palliative care
PC: Palliative care
HM: Hematologic malignancy
