# Supplementary material for: Assessing palliative care needs in adult patients with hematological malignancies and their caregivers: implications for referral practice
Source: BMC Palliat Care. 2025 Jul 14;24:201. doi: 10.1186/s12904-025-01811-5 (PMC12261735; doi:10.1186/s12904-025-01811-5)
Supplement: Supplementary file 1 — Supplementary Material 1 [file 12904_2025_1811_MOESM1_ESM.pdf]

Supplement I

NEEDS ASSESSMENT TOOL: PROGRESSIVE DISEASE CANCER (NAT: PD-C)

COMPLETE ALL SECTIONS

**Patient Name:**	<<Patient Full Name>>
**Diagnosis:**	<<Patient Diagnosis>>
**Date:**	<<Date>>

SECTION 1: PRIORITY REFERRAL FOR FUTHER ASSESSMENT

Please mark with an 'X' relevant columns	**Yes**	**No**	
**1. Does the patient have a caregiver readily available if required?**		•	**If dotted boxes are ticked, consider assessment by SPCS**
**2. Has the patient or caregiver requested a referral to a Specialist Palliative Care Service?**	•		
**3. Do you require assistance in managing the care of this patient and/or family?**	•		

SECTION 2: PATIENT WELLBEING

Record level of concern for each item by marking relevant column with an 'X'.
If a need is identified (ie "some/potential" or "significant" level of concern), record an action to address this need.
If a referral is required for any item, please complete the referral section at the end of this form.

	**Level of Concern**			**Action Taken**		
	None	Some/ Potential	Significant	Directly managed	Managed by other care team member	Referral required (complete referral section at end of form)
1. Is the patient experiencing unresolved physical symptoms (including problems with pain, sleeping, appetite, nausea, bowels, breathing or fatigue)?						
2. Does the patient have problems with daily living activities?						
3. Are the patient's psychological symptoms interfering with wellbeing or relationships?						
4. Does the patient have concerns about spiritual or existential issues?						
5. Does the patient have financial or legal concerns that are causing distress or require assistance?						
6. From the health delivery point of view, are there health benefits, cultural or social factors involving the patient or family that are making care more complex?						

7. Does the patient require information about (please delete irrelevant options):

- ☐ Their prognosis
- ☐ The cancer
- ☐ Treatment options
- ☐ Financial/legal issues
- ☐ Medical/health/support services
- ☐ Social/emotional issues

COMMENTS:

9

10

SECTION 3: ABILITY OF CAREGIVER OR FAMILY TO CARE FOR PATIENT

Record level of concern for each item by marking relevant column with an 'X'.
If a need is identified (ie "some/potential" or "significant" level of concern), record an action to address this need.
If a referral is required for any item, please complete the referral section at the end of this form.

**Who provided this information? (please delete irrelevant options) ☐ Patient ☐ Caregiver ☐ Both**	**Level of Concern**			**Action Taken**		
	None	Some/ Potential	Significant	Directly managed	Managed by other care team member	Referral required (complete referral section at end of form)
1. Is the caregiver or family distressed about the patient's physical symptoms?						
2. Is the caregiver or family having difficulty providing physical care?						
3. Is the caregiver or family having difficulty coping?						
4. Does the caregiver or family have financial or legal concerns that are causing distress or require assistance?						
5. Is the family currently experiencing problems that are interfering with their functioning or inter-personal relationships, or is there a history of such problems?						

6. Does the caregiver or family require information about (please delete irrelevant options):

☐ Their prognosis
☐ The cancer
☐ Treatment options
☐ Financial/legal issues
☐ Medical/health/support services
☐ Social/emotional issues

COMMENTS:

11

SECTION 4: CAREGIVER WELLBEING

Record level of concern for each item by marking relevant column with an 'X'.
If a need is identified (ie "some/potential" or "significant" level of concern), record an action to address this need.
If a referral is required for any item, please complete the referral section at the end of this form.

**Who provided this information? (please delete irrelevant options) ☐ Patient ☐ Caregiver ☐ Both**	**Level of Concern**			**Action Taken**		
	None	Some/ Potential	Significant	Directly managed	Managed by other care team member	Referral required (complete referral section at end of form)
1. Is the caregiver or family experiencing physical, practical, spiritual, existential or psychological problems that are interfering with their wellbeing or functioning?						
2. Is the caregiver or family experiencing grief over the impending or recent death of the patient that is interfering with their wellbeing or functioning?						

COMMENTS:

12

IF REFERRAL(S) REQUIRED FOR FUTHER ASSESSMENT OR CARE, PLEASE COMPLETE THIS SECTION

1. Referrals suggested to be made (please note disciplines required, eg. community nurse, home care etc...:

2. Priority of assessment needed:
(Please delete irrelevant options)

- ☐ Urgent (within 24 hours)
- ☐ Semi-Urgent (2-7 days)
- ☐ Non-Urgent (next available)

3. Discussed the referral with the client (please delete irrelevant option) **Yes / No**

4. Client consented to the referral (please delete irrelevant options) **Yes / No**

5. Referral from:

Name: <<Doctor: Name>>

Signature: ____________________________

Needs Assessment Tool Copyright Notice
© Centre for Health Research and Psycho-oncology (2009) NAT:PD-C.
Acknowledgement: NSW Central West Division of General Practice for their assistance in developing this template.
